# Potential Role of Porcine Reproductive and Respiratory Syndrome Virus Structural Protein GP2 in Apoptosis Inhibition

**DOI:** 10.1155/2014/160505

**Published:** 2014-01-09

**Authors:** Sujit Pujhari, Tayyba T. Baig, Alexander N. Zakhartchouk

**Affiliations:** ^1^International Vaccine Center (VIDO-InterVac), Vaccine and Infectious Disease Organization, University of Saskatchewan, 120 Veterinary Road, Saskatoon, SK, Canada S7N 5E3; ^2^Department of Microbiology and Immunology, University of Saskatchewan, Saskatoon, SK, Canada S7N 5E5

## Abstract

Porcine reproductive and respiratory syndrome virus (PRRSV) is a serious threat to the pork industry, and its pathogenesis needs further investigations. To study the role of two structural proteins of PRRSV in virus-host cells interactions, two stable cell lines (MARC-2a and MARC-N) expressing GP2 and N proteins, respectively, were established. We induced apoptosis in these cells by treating them with staurosporine and found a significant reduction in the number of apoptotic cells in MARC-2a as compared to MARC-N and MARC-145 cells. In addition, we found significantly higher activities of transcriptional factors (NF-**κ**B and AP-1) in both cell lines as compared to MARC-145 (parent cells). Overall, our data suggest that, although both stable cell lines activate NF-**κ**B and AP-1, GP2 triggers the antiapoptotic process through an intermediate step that needs to be further investigated.

## 1. Introduction

Porcine reproductive and respiratory syndrome virus (PRRSV) along with Lactate dehydrogenase-elevating virus, Equine arteritis virus, and Simian hemorrhagic fever virus belongs to the family *Arteriviridae* of order *Nidovirales*. It causes one of the most economically significant diseases in the swine industry. In PRRSV, the full-length single stranded genomic RNA is almost 15.4 kb in length with a 5′ cap and 3′ polyadenylation. It encodes ten ORFs (1a, 1b, 2a, 2b 3, 4, 5a, 5b, 6, and 7), flanked by 5′ and 3′ untranslated regions [1, 2]. ORF 1a and 1b constitute the majority of the genome and encode viral nonstructural proteins while the rest codes for structural proteins: GP2, E, GP3, GP4, GP5, 5a, M, and N. A lipid bilayer envelope surrounds the viral RNA embedded within nucleocapsid (N) [3]. Of the structural proteins, GP2, GP3, GP4, and GP5 are glycosylated and present on the viral envelope, along with the nonglycosylated M and E proteins. GP5 is known as the major envelope glycoprotein, based on its abundance in the virion, whereas the GP2, GP3, and GP4 are the minor envelope glycoproteins [1]. The 3′-proximal genome part has a compact organization, and most of the genes have overlapping sequences. For instance, ORF 2b, encoding the E protein, is partially overlapping ORF 2a that encodes the GP2 protein.

Apoptosis is an important mechanism by which virus-infected cells are eliminated from the host; therefore, many viruses have evolved strategies to prevent or delay apoptosis in order to provide a window of opportunity in which virus replication, assembly, and egress can take place. Interfering with apoptosis may also be required for establishment and/or maintenance of persistent infections.

PRRSV induces apoptosis both *in vitro* and *in vivo* [4–6]. However, it is debatable whether PRRSV induces apoptosis directly (in infected cells) or indirectly (in bystander cells). Lee and Kleiboeker [7] demonstrated that PRRSV induced apoptosis in infected MARC-145 cells through a mitochondria-mediated pathway. In addition, Costers and colleagues [8] showed that both anti- and proapoptotic activities take place in PRRSV-infected MARC-145 cells and macrophages. It appears that, early in infection, PRRSV stimulates antiapoptotic pathways, whereas infected cells die by apoptosis later.

Previous studies demonstrated that PRRSV infection causes an activation of NF-*κ*B and AP-1 transcription factors [9–11]. Moreover, the ERK signaling pathway is also activated in PRRSV-infected cells [12, 13], and activation of JNK is required for the virus-induced apoptosis [14]. However, the molecular mechanisms behind these events are poorly understood. Previous studies showed that PRRSV N [11, 15] and nonstructural protein 2 [16] contribute to NF-*κ*B activation. In the present study, we investigated the role of the PRRSV GP2 and N proteins in apoptosis inhibition and NF-*κ*B and AP-1 signaling pathways activation using stable cell lines MARC-2a and MARC-N, expressing the GP2 and N proteins, respectively.

## 2. Materials and Methods

### 2.1. Cells

MARC-145 (MA-104 clone, African green monkey kidney cell line) cells were maintained in minimal essential medium (Hyclone) supplemented with 10% fetal bovine serum (FBS), 10 mM HEPES, 10 mM nonessential amino acids, and 100 U of gentamycin.

### 2.2. Antibodies

NF-*κ*B rabbit monoclonal antibody (MAb) and c-Jun mouse MAb were purchased from Cell Signaling Technology. Anti-N mouse MAb (SDOW-17) was purchased from Rural Technologies. Fibrillarin mouse MAb was purchased from Santa Cruz. Alpha-tubulin antibody was purchased from Sigma-Aldrich. Cy2-conjugated AffiniPure goat anti-mouse and goat anti-rabbit immunoglobulin G (IgG) were purchased from Jackson ImmunoResearch. Horseradish peroxidase (HRP) conjugated goat anti-mouse IgG and goat anti-rabbit IgG were purchased from Bio-Rad.

### 2.3. Generation of Stable Cell Lines

Synthesis of the codon-optimized N gene (PRRSV strain VR2332) was ordered from EZBiolab. A 380 bp fragment, amplified by primers (N-FOR-B-CG*ggatcc*ATGCCAAATAATAATGGTAA and N-REV-B-CG*ggatcc*CTAAGCGGATGGAGAAGCAG) was digested with *Bam*HI and inserted into *Bam*HI site of the plasmid pIREShyIA [17] creating pIREShyIA-N.

The pUC57-GP2 plasmid containing a codon-optimized sequence for GP2 gene (PRRSV strain VR2332) was ordered from GenScript and the pIREShyIA-GP2 plasmid for the eukaryotic expression of GP2 protein was constructed as follows: first, a 780 bp fragment was amplified using primers containing *Bam*HI sites (sORF2pUC57-AT*ggatcc*ATGAAGTGGGGCCCCTG and asORF2pUC57-AT*ggatcc*TCACTGGGAGTTG) and pUC57-GP2 plasmid DNA as a template. Second, this fragment was digested with *Bam*HI and inserted into *Bam*HI sites of pIREShyIA vector, creating pIREShyIA-2a. Note, natural GP2 gene (ORF 2a) contains an additional ORF (ORF 2b) encoding E protein. To avoid the expression of E protein, the initiation codon of ORF 2b in the synthetic GP2 gene was mutated from ATG to GTG.

To produce the stable cell lines (MARC-2a and MARC-N), subconfluent monolayers of MARC-145 cells were transfected with DNAs of pIREShyIA-2a and pIREShyIA-N, respectively, using Lipofectamine-2000 reagent (Invitrogen) and cell clones were selected by hygromycin B (300 *μ*g/mL).

### 2.4. NF-*κ*B and AP-1 SEAP Reporter Assay

To monitor the activation of NF-*κ*B and AP-1 signal transduction pathways, the plasmids (pNF*κ*B-SEAP or pAP1-SEAP) were purchased from Clontech and were transiently transfected into MARC-145, MARC-2a, or MARC-N cells by the use of Lipofectamine LTX (Invitrogen). Briefly, 4 *μ*g of each plasmid DNA, 12 *μ*L lipofectamine LTX, and 4 *μ*L Plus reagent were used to transfect cells, seeded in a well of 6-well plate. Since these transfected plasmids contain the secreted alkaline phosphatase (SEAP) gene as a reporter, culture supernatants were collected 72 h after transfection, and SEAP activity was detected in by the Great Escape SEAP Chemiluminescence Assay kit (Clontech) using a GloMax 20/20 Luminometer (Promega). The chemiluminescence emitted by a SEAP-activated substrate (CSPD) was measured in relative luminescence units.

### 2.5. Nuclear and Cytoplasmic Fractionation

MARC-145, MARC-2a, and MARC-N cells were seeded (1 × 10^6^ per well) on 6-well plates. Next day, cells on the plates were washed twice with ice-cold phosphate-buffered saline (PBS) before being scrapped and collected at 1000 rpm for 10 minutes. Cells were resuspended in 450 *μ*L buffer A (100 mM HEPES, pH 7.9, 100 mM KCl, 0.5 mM EDTA, 0.1 mM EGTA, 1 mM dithiothreitol [DTT], and 1 mM phenylmethylsulfonyl fluoride [PMSF]), allowed to swell on ice for 30 minutes, and vortexed for three times/30 s each. Extracts were collected by centrifugation at 14,000 rpm for 10 min and the supernatant was the cytoplasmic fraction. To remove any residual cytoplasmic extracts contamination, cells were washed using buffer A, followed by PMSF added PBS. The pellet was then resuspended and incubated for 20 minutes in 100 *μ*L buffer B (200 mM HEPES, pH 7.9, 500 mM KCl, 5.0 mM EDTA, 1.0 mM EGTA, 1 mM DTT, and 1 mM PMSF) and centrifuged at 14,000 rpm for 5 min at 4°C. Now, the supernatant was the nuclear fraction. Protein concentrations in both fractions were estimated by Bradford assay, and the purity of the fractions was tested by Western blotting for tubulin and fibrillarin to define the cytoplasmic and nuclear fractions, respectively. Equal amounts of nuclear fractions were analyzed to allow the comparison of protein expression levels in different cells.

### 2.6. Western Blot Analysis

Denatured protein samples prepared from nuclear and cytoplasmic fractions were resolved by sodium dodecyl sulfate-10% polyacrylamide gel electrophoresis (SDS-10% PAGE) and transferred to nitrocellulose membrane. To block nonspecific binding sites on the membrane, it was placed over Tris-buffered saline (0.1 M Tris [pH 7.6], 0.9% NaCl) containing 0.1% Tween 20 and 5% skim milk for 1 h at room temperature. The membranes were incubated at 4°C overnight with appropriate primary antibodies diluted (1 : 1000) in 5% bovine serum albumin (BSA) or 1% skim milk prepared in 0.1% TBST as per manufacturer's instructions. HRP-labeled anti-rabbit IgG or anti-mouse IgG detection antibodies diluted (1 : 2000) in 1% skim milk prepared in 0.1% TBST were then added at room temperature for 1 h and signals were detected with the enhanced chemiluminescence method (Bio-Rad). The band intensities were measured densitometrically. The band intensities data were normalized with fibrillarin and MARC-145 value was adjusted to 1 for comparison and data were represented as fold changes. For this, the unsaturated bands on the X-ray films were scanned and saved as 8-bit grayscale JPEG files and were analyzed by using the public domain software ImageJ from the National Institutes of Health.

### 2.7. Immunofluorescence Staining

MARC-N and MARC-145 cells at 85–95% confluency in 2-well Lab-Tek chamber slides were fixed with absolute methanol for 10 min at −20°C. After rehydration with PBS, cells were incubated with anti-N monoclonal antibody SDOW-17 (dilution 1 : 500) for 1 h at room temperature. Cells were rinsed three times with PBS and incubated with Cy2-conjugated AffiniPure Goat Anti-Mouse IgG (dilution 1 : 200). The cells were examined using a Zeiss Axiovert 200 M inverted fluorescent microscope.

### 2.8. Assessment of Apoptosis Using Annexin V/PI and Hoechst Staining

A commercially available annexin V apoptosis detection kit (Invitrogen) and flow cytometry were used to determine the annexin V-binding cells. Appropriate cells were grown on 35 mm disc. Set of wells were treated with 1 *μ*M staurosporine (an apoptotic inducer), for 24 h, keeping another set as an untreated control. After collecting and washing twice with PBS, treated or untreated cells were resuspended in the binding buffer (500 *μ*L), followed by the addition of FITC-annexin-V (5 *μ*L) PI (5 *μ*L) sequentially. The samples were then incubated for 15 min in the dark at room temperature and subjected to flow cytometric evaluation. The experiment was performed in triplicate and repeated three times.

Nuclear morphology of control and staurosporine treated cells was observed by staining cell nuclei with Hoechst 33342 (Invitrogen). Briefly, cells were incubated with Hoechst 33342 (10 *μ*g/mL) for 15 min at RT and examined and counted manually (at least 200 per slide) under a fluorescence microscope by using the DAPI filter. Apoptotic cells were characterized by the condensation of chromatin and/or nuclear fragmentation.

### 2.9. MTT Assay

MARC-145, MARC-2a, or MARC-N cells were split up into 96-well plates at a density of 10^5^ cells per well. Next day, staurosporine (3 *μ*M) was added. After 24 h, the 3-(4,5-dimethylthiazol-2-yl)-2,5-diphenyl-2H-tetrazolium bromide (MTT) assay for mitochondrial activity, an indicator of cell viability, was carried out by adding 25 *μ*L of MTT (5 mg/mL) to each well and incubating the cells for 2 h in a CO_2_ incubator at 37°C. Finally, 100 *μ*L of lysis buffer (20% SDS in 50% dimethylformamide, pH 4.7) was added, and the cells were further incubated overnight before measuring the optical density at A_595_ with an ELISA reader. The percentage of living cells was calculated using the following formula: (A_595_ treated cells)/(A_595_ nontreated cells) ×100. The assay was performed in triplicate, and the experiment was repeated three times.

### 2.10. Statistical Analysis

All data were analyzed using the GraphPad Prism (Version 5.03) software. Differences among all groups were examined using the one-way ANOVA followed by Tukeys test. Differences were considered significant if *P* < 0.05.

## 3. Results

### 3.1. Construction of MARC-2a and MARC-N Cell Lines

To analyze molecular and pathological roles of GP2 and N proteins, we constructed two stable cell lines MARC-2a and MARC-N, expressing PRRSV GP2 and N proteins, respectively. The parent cell line used for this purpose was MARC-145 cell line because it supports infection and propagation of PRRSV.

**Figure 1:**
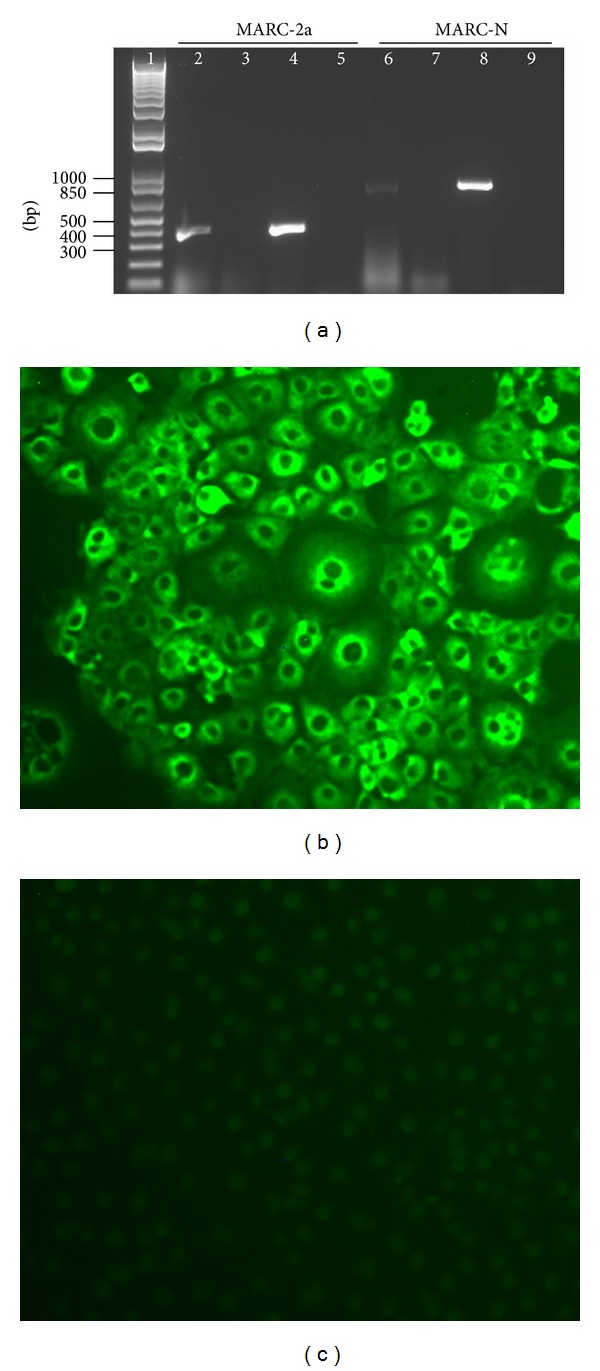
Characterization of GP2 and N expressing stable cell lines. (a) Agarose gel analysis. DNA was extracted from both cell lines and subjected to PCR amplification with primers specific to the 2a and N genes. Template amplification with no DNase and no reverse transcription (RT) treatment (lanes 2 and 6); template amplification with DNase treatment and no RT (lanes 3 and 7); template amplification with DNase and RT treatment (lanes 4 and 8); no template controls (lanes 5 and 9). Lane 1 represents the GeneRuler 1 kb Plus DNA Ladder from Fermentas. (b) Immunofluorescence staining of MARC-N cells to show evidence of the N protein expression. (c) Immunofluorescence staining of MARC-145 parent cells.

The plasmids pIREShyIA-2a and pIREShyIA-N contained the gene sequences for GP2 and N, respectively, under the control of the human cytomegalovirus promoter, and they also contained the hygromycin B phosphotransferase gene as a selectable marker fused to the internal ribosome entry site (IRES) sequence at the 5′ end. The IRES permits the translation of two open reading frames from one mRNA. MARC-145 cells were transfected with these engineered plasmids and grown under the selection pressure of hygromycin B. Hygromycin-resistant cell clones were further expanded and maintained in the presence of hygromycin B for several days. Cells from each of the maintained clones (MARC-2a and MARC-N) were used for the extraction of total nucleic acids. PCR analysis with the primers specific to the 2a and N genes revealed the amplified products of 380 bp and 780 bp, respectively (Figure 1(a), lanes 2 and 6). To confirm the presence of mRNA corresponding to these genes in the cells, the nucleic acids were first treated with DNase, and then an aliquot of each reaction was used as a template for cDNA synthesis followed by PCR amplification (Figure 1(a), lanes 4 and 8). Another aliquot from each reaction was used directly as a template for PCR (Figure 1(a), lanes 3 and 7) to rule out the possibility that the amplified products, shown in lanes 4 and 8, were from DNA and not from mRNA.

Following the confirmation of mRNA expression, the expression of N-gene at the protein level was evidenced by immunofluorescence staining only in MARC-N cells (Figure 1(b)) and not in parent MARC-145 cells (Figure 1(c)). For 2a gene, we could not show protein expression due to the unavailability of the GP2-specific antibody.

### 3.2. Evaluation of Apoptosis Inhibition

**Figure 2:**
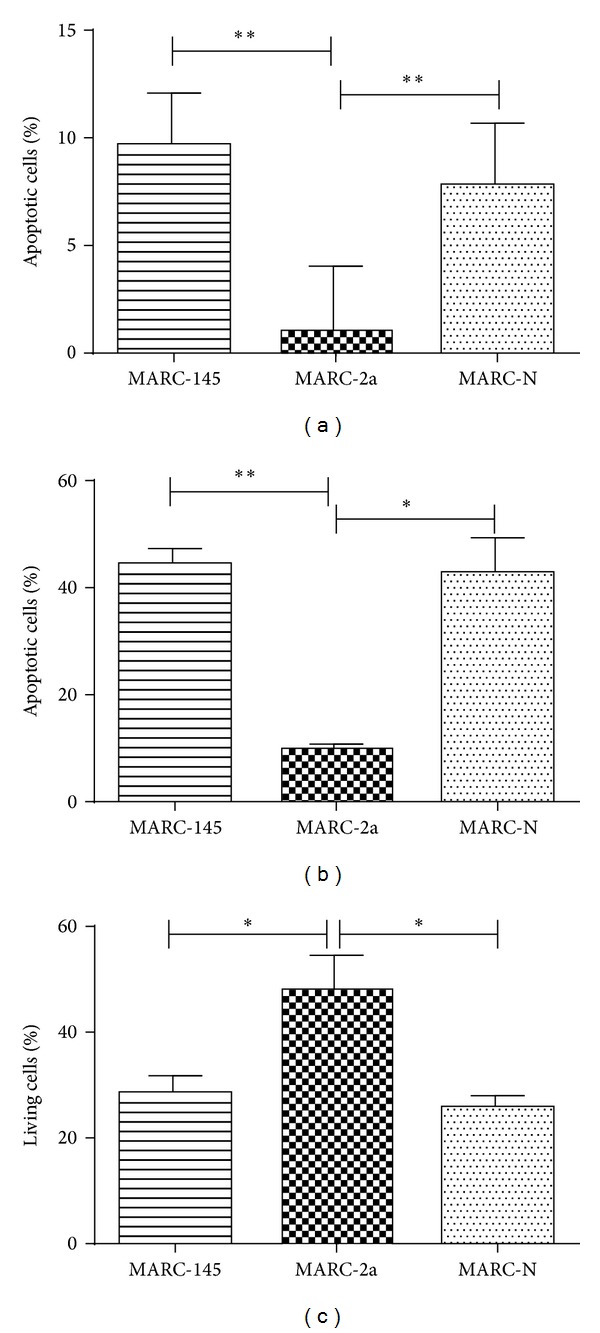
Inhibition of apoptosis in MARC-2a cells. After incubation for 24 h with 1 *μ*M staurosporine, MARC-145, MARC-2a, and MARC-N cells were stained with FITC-annexin V and PI (a) or Hoechst 33342 (b). The percentage of viable cells was determined by MTT assay (c). The data represent the results of three independent experiments in triplicates, and error bars indicate standard deviations of the means. *P* < 0.05 (*); *P* < 0.01 (**).

To study apoptosis inhibition in stable cell lines, MARC-145, MARC-2a, and MARC-N cells were stained with annexin V-FITC and PI and analyzed by FACS to detect and quantitatively determine the percentage of dead, viable, apoptotic, and necrotic cells. To induce apoptosis, the cells were treated with staurosporine and analyzed at 24 h after treatment (Figure 2(a)). We found a significantly low percentage of MARC-2a cells (1.5%) that were positive for annexin V-FITC (apoptotic) in comparison to MARC-145 (9.5%) and MARC-N (8.3%) cells (*P* < 0.05).

For further verification of these findings, staurosporine treated cells were stained with Hoechst. This nuclear stain allowed discriminating normal live cells (blue homogenate fluorescence) from apoptotic cells with fragmented nucleus and condensed chromatin material (brighter granulated blue color fluorescence). In agreement with the annexin V staining, MARC-2a cells had significantly lower percentage of apoptotic cells (Figure 2(b)).

Finally, after treatment with staurosporine, the percentage of viable MARC-2a cells was higher than the percentage of living cells in MARC-145 and MARC-N cell lines (Figure 2(c)).

Taken together, these data indicate that MARC-2a cells are more resistant to programmed cell death than to parent MARC-145 cells or MARC-N cells.

### 3.3. NF-*κ*B and AP-1 Activation

NF-*κ*B and AP-1 activation in MARC-2a and MARC-N cells was confirmed using the SEAP reporter gene system. The principal behind this assay is the following. If the NF-*κ*B or AP-1 signal transduction pathway is induced, endogenous NF-*κ*B/AP-1 binds to the k/Ap enhancer element, located in the promoter region of the pNF*κ*B-SEAP or pAP1-SEAP vector, thus activating the transcription of the SEAP reporter gene.

**Figure 3:**
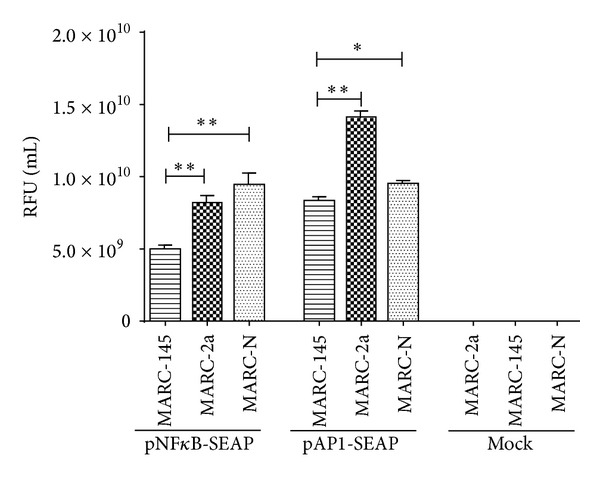
NF-*κ*B and AP-1 activity in MARC-145, MARC-2a, and MARC-N cells. Cells were transfected with the secreted alkaline phosphatase (SEAP) reporter vectors: pNF*κ*B-SEAP and pAP1-SEAP. The culture supernatants were collected and analyzed for SEAP activity and represented as relative fluorescence unit (RFU) per mL. The data represent the results of three independent experiments in triplicates, and error bars indicate standard deviations of the means. *P* < 0.05 (*); *P* < 0.01 (**).

As shown in Figure 3, MARC-2a and MARC-N cells had significantly increased levels of SEAP than MARC-145 cells, which indicate the NF-*κ*B and AP-1 activation in these cells. Untransfected cells did not show any SEAP activity.

**Figure 4:**
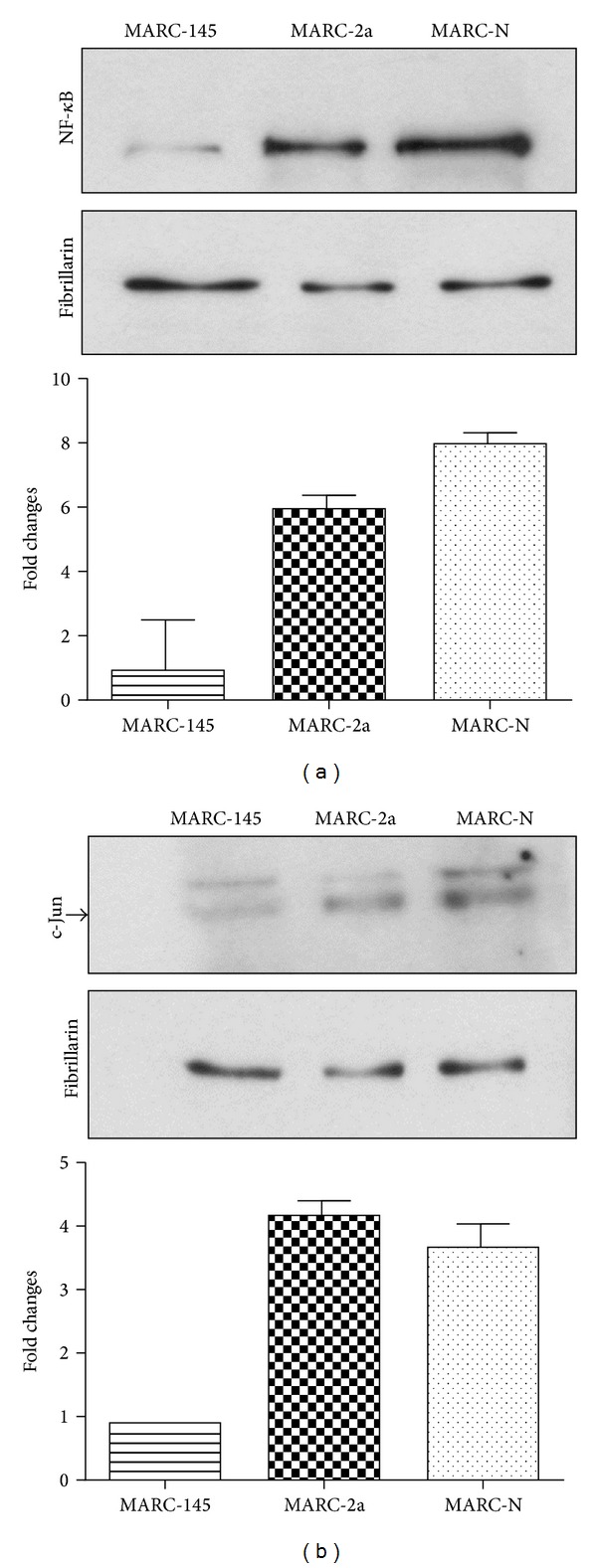
Western blot analysis of NF-*κ*B and AP-1 activation. Equal amounts of nuclear protein fractions were separated on 10% SDS-PAGE. Cytoplasmic fraction contamination was evaluated by *α*-tubulin antibody (data not shown). Fibrillarin antibody was used as a nuclear marker and also as a loading control. Fold changes in protein amounts are plotted. Western blot with (a) NF-*κ*B rabbit monoclonal antibody and (b) c-Jun mouse monoclonal antibody. Arrow shows the position of the 48 kDa band corresponding to the phosphorylated form of the c-Jun protein.

Additionally, these data were confirmed by Western blot analysis (Figure 4). For biological functions, transcription factors have to go to the nucleus. Thus, we have analyzed the nuclear fractions of MARC-145, MARC-2a and MARC-N cells for the presence of NF-*κ*B and c-Jun (part of AP-1) proteins. Fibrillarin, the nucleolar protein, was used as the loading control. From ImageJ analysis, there was found a 6-fold and 8.5-fold increase in the expression of NF-*κ*B in MARC-2a, and MARC-N cells, respectively, as compared to MARC-145 cells (Figure 4(a)). In case of AP-1, the fold increase was 4.5 and 4, respectively (Figure 4(b)).

## 4. Discussion

Many viruses, including PRRSV, utilize strategies of delaying the process of apoptosis for their benefit. Apoptosis can be triggered by a variety of stimuli including death receptors on the cell surface (extrinsic pathway) and intracellular signals (intrinsic pathway). Staurosporine, a strong inhibitor of protein kinases [18], triggers intrinsic pathway which results in the release of cytochrome c from mitochondria, formation of the apoptosome (cytochrome c, APAF-1, and caspase-9), and activation of caspase-9. Both the extrinsic and the intrinsic processes congregate at the activation of the downstream effector caspases, which are responsible for inducing the morphological changes observed in an apoptotic cell. The apoptotic events are regulated by the interplay of pro- and antiapoptotic proteins which are members of the Bcl-2 family [19].

Many viruses encode homologue of Bcl-2 proteins that can prevent apoptosis, thus helping the viruses to complete their life cycle in the host cells [20]. For instance, human adenovirus E1B 19 K protein inhibits apoptosis by forming heterodimers with a variety of proapoptotic proteins of the Bcl-2 family [21]. However, our attempts to detect interactions between GP2 and proapoptotic members of the Bcl-2 family were not successful (data not shown).

On the other hand, the cell survival in many viral infections is regulated by the NF-*κ*B activity. For instance, expression of the human hepatitis C core protein activates the NF-*κ*B pathway which results in an antiapoptotic activity [22]. Also, interferon (IFN) signaling activates the NF-*κ*B that integrates into the IFN receptor pathways and promotes cell survival [23]. It is also proved that IL-15 has an antiapoptotic effect [24, 25]. The antiapoptotic property of IL-15 and its regulation by NF-*κ*B signaling in PRRSV infection hinted a possible association with the cell survival strategy of PRRSV. On the other hand, AP-1 has an evolutionary origin from the avian sarcoma virus with cell proliferation and differentiation property that support its role in cell survival signaling [26].

## 5. Conclusions 

The main goal of our research is to understand the role of the PRRSV structural proteins in the viral pathogenesis. In the current study, we have explored the role of PRRSV GP2 and N proteins in activation of the NF-*κ*B and AP-1 signaling pathways. The role of the N protein in activation of NF-*κ*B has been established earlier, whereas activation of cell genes transcription by GP2 has never been previously reported. To our best knowledge, this study is the first report, describing genes transcription activation function of GP2 protein. We have also demonstrated that N activates the AP-1 pathway in addition to NF-*κ*B, which is a novel finding too.

The GP2 protein likely plays a role in apoptotic inhibition by PRRSV. Possibly, the transcription factors activated by GP2 protein are involved in this activity by activation of antiapoptotic and suppression of proapoptotic genes expression. However, the mechanism of this GP2 function needs to be further investigated.
